# Data-Driven Optimization of Piezoelectric Energy Harvesters via Pattern Search Algorithm

**DOI:** 10.3390/mi12050561

**Published:** 2021-05-15

**Authors:** Yang Huang, Zhiran Yi, Guosheng Hu, Bin Yang

**Affiliations:** 1 National Key Laboratory of Science and Technology on Micro/Nano Fabrication, Shanghai Jiao Tong University, Shanghai 200240, China; cedarhuang@sjtu.edu.cn (Y.H.); yizhiran@sjtu.edu.cn (Z.Y.); huguosheng2019@sjtu.edu.cn (G.H.); 2 Department of Micro/Nano Electronics, Shanghai Jiao Tong University, Shanghai 200240, China; 3 State Key Laboratory of Mechanical System and Vibration, School of Mechanical Engineering, Shanghai Jiao Tong University, Shanghai 200240, China

**Keywords:** piezoelectric, energy harvester, optimization, pattern search, FEM, PZT

## Abstract

A data-driven optimization strategy based on a generalized pattern search (GPS) algorithm is proposed to automatically optimize piezoelectric energy harvesters (PEHs). As a direct search method, GPS can iteratively solve the derivative-free optimization problem. Taking the finite element method (FEM) as the solver and the GPS algorithm as the optimizer, the automatic interaction between the solver and optimizer ensures optimization with minimum human efforts, saving designers’ time and performing a more precise exploration in the parameter space to obtain better results. When employing it for the optimization of PEHs, the optimal length and thickness of PZT were 6.0 mm and 4.6 µm, respectively. Compared with reported high-output PEHs, this optimal structure showed an increase of 371% in output power, an improvement by 1000% in normalized power density, and a reduction of 254% in resonant frequency. Furthermore, Spearman’s rank correlation coefficient was calculated for evaluating the correlation among geometric parameters and output performance such as resonant frequency and output power, which provides a data-based perspective on the design and optimization of PEHs.

## 1. Introduction

With the urgent demand of sustainable power supplies for low-power electronic applications such as wireless sensor network systems [1,2,3,4] in Internet of Things, implantable medical devices [5,6] and other devices in some extreme environments [7], energy harvesting from the ambient environments has attracted broad attention and provided potential solutions to the periodical replacement of batteries during the last few decades. Vibration energy is ubiquitous and robust mechanical energy that exists widely, including bridges [8], roads [9], human body [10,11,12], cars [7], etc. Therefore, a variety of vibration-based piezoelectric energy harvesters (PEHs) with different structures have been proposed and studied [13,14,15]. Among these, the cantilever structure is widely used due to structure simplicity and the high average strain obtained by a given input force [2,16,17,18,19].

For cantilever PEHs, a multi-parameter coupling problem exists for obtaining high-efficiency energy conversion. To improve the output performance of cantilever PEHs, researchers have studied the effects of different geometric parameters on output performance [20,21]. He et al. [22] and Jia et al. [23] proposed that the optimal mass-beam length ratio is 0.6~0.7 within linear response. Hu et al. [18] investigated the optimal length of the piezoelectric layers based on theoretical analysis, FEM simulation and experimental verification, from which they discovered that the optimal length ratio of piezoelectric layers and the beam is approximately 0.2. Furthermore, Hu et al. [18] also reported that the optimal PZT layout length decreases as cantilever width increases. However, investigations on various geometric parameters in the studies above constitute single-variable optimization. Moreover, the geometric parameters were manually set and tested at a fixed interval based on the researchers’ experience and intuition, which required the researcher to spend more time in trying each possible combination of geometric parameters to obtain the optimal output performance. To overcome these shortcomings, several data-driven optimization strategies combining different algorithms to maximize the output performance of PEHs have been proposed recently. For example, data-driven optimization can solve the optimization problems that are difficult to formulate or solve, in that they are non-convex, multi-objective or multi-modal; it can solve optimization problems based on derivative-free data. Ghoddus et al. [24] presented an optimization approach based on the Particle swarm optimization (PSO) algorithm to maximize the output power of PEHs with four different structures, which simultaneously optimized multiple geometric parameters. In addition, Nabavi and Zhang [25,26] proposed an analytical model for PEHs with proof mass and used a genetic algorithm to simultaneously optimize multiple objectives, including resonant frequency, output power and device volume. However, finding the optimal solution to complex high-dimensional problems such as the multi-parameter coupling problem for PEHs may require expensive objective function evaluations. Therefore, as population-based stochastic optimization techniques, the PSO and GA algorithm implemented in the previous works [24,25,26] may encounter low efficiency when dealing with the multi-parameter coupling problems. Moreover, parameter tuning is needed to obtain a better convergence for the PSO algorithm and GA [27], such as particle number, accelerate constant, inertia weight and population size. Although the global search ability of GA and PSO may be better, the generalized pattern search (GPS) algorithm is more suitable for the time-consuming multi-parameter coupling problem due to its effortless parameter tuning and relatively fewer objective function evaluations [28].

In this paper, we proposed a data-driven optimization strategy based on the GPS algorithm. The GPS algorithm is one of the direct search methods, which can be effectively used in derivative-free optimization. By implementing the proposed scheme, multiple geometric parameters ($l_{p},\;w,\;t_{p}$, and $t_{b}$) were simultaneously optimized for maximum output performance based on the data without building an analytical model. Using the proposed optimization method can not only efficiently optimize the geometric parameters of PEHs to achieve high output performance but also save time for analytical modeling and complex parameter tuning. Furthermore, Spearman’s rank correlation coefficient was calculated for evaluating the correlation among geometric parameters and output performance, providing a data-based perspective on the design of PEHs.

## 2. Methods

**Figure 1:**
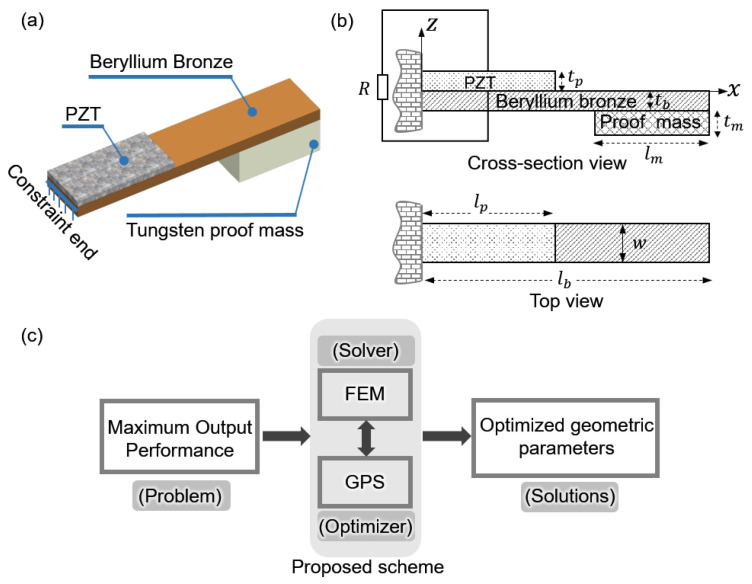
Schematic diagram of the piezoelectric energy harvester (PEH) and the proposed strategy. (**a**) Three-dimensional view. (**b**) Annotation for geometric parameters of the cantilever PEH. (**c**) Overview of the presented optimization working process.

The unimorph PEH is composed of a piezoelectric layer, a structural layer, and a proof mass. The piezoelectric layer partially covers the flexible structural layer along the constraint end, as shown in Figure 1a. A tungsten proof mass is fixed at the free end of the structural layer, increasing average strain under given excitation and lowering the resonant frequency to meet the low-frequency ambient vibration. Figure 1b illustrates the annotation of this unimorph PEH. The data-driven optimization scheme mainly includes a FEM solver and a GPS optimizer. Figure 1c depicts the overall function of the presented strategy. In this paper, FEM simulation was carried out using COMSOL Multiphysics (Version 5.4) and the material properties used in FEM simulation are listed in Table 1. The GPS algorithm was implemented in MATLAB (Version 2020a). The communications between solver and optimizer were implemented using COMSOL LiveLink^TM^ for the MATLAB module, which facilitated the data-driven optimization strategy. As direct search methods, different types of pattern search algorithms have been utilized for solving engineering optimization problems [28,29,30]. The convergence analysis of GPS algorithm has been performed by Audet and Dennis [31].

At each iteration, the GPS searches a set of points around the current point called mesh, finding a better point whose value of the objective function is lower than the value before. Then, the better point is set as the current point at the next iteration. Based on this procedure (Polling), the GPS finds a sequence of points that approaches the optimum, and it does not stop until the convergence is met. Specifically, let $M_{k}$ denote the mesh at *k*-th iteration, and $x_{k}^{\left(i\right)}$ denote the *i*-th point in the $M_{k}$. The mesh is defined as Equation (1):

(1) $$M_{k}≜\left\{x\in {\mathbb{R}}^{n}|x=x_{k}^{\prime }+\Delta_{k}\cdot \nu_{k},\;k\in \left\{1,2,\cdots ,\;n\right\}\right\}$$

where $x_{k}^{\prime }$ denotes the current point at the *k*-th iteration while $\Delta_{k}$ denotes mesh size, and $\nu_{k}$ denotes pattern. Pattern is a set of vectors used to determine the generation of mesh. Suppose F(x) denotes the objective function in the optimization problem. At each iteration, GPS computes $F\left(x_{k}^{\left(i\right)}\right)$ and looks for a better point $x_{k}^{\left(j\right)}$ so that $F\left(x_{k}^{\left(j\right)}\right)<F(x_{k}{}^{\prime })$; then, $x_{k+1}^{\prime }=x_{k}^{\left(j\right)}$ and $\Delta_{k+1}=2\cdot \Delta_{k}$ are set. Otherwise, if the polling fails to find a better point at $k^{th}$ iteration, then $x_{k+1}^{\prime }=x_{k}^{\prime }$ and $\Delta_{k+1}=1/2\cdot \Delta_{k}$. The computation in the mesh at each iteration is called polling. In addition, the search method runs before polling can select a different current point, which may accelerate the optimization if tuned well. Various search methods can be set, including the genetic algorithm, Latin hypercube search, etc.

**Figure 2:**
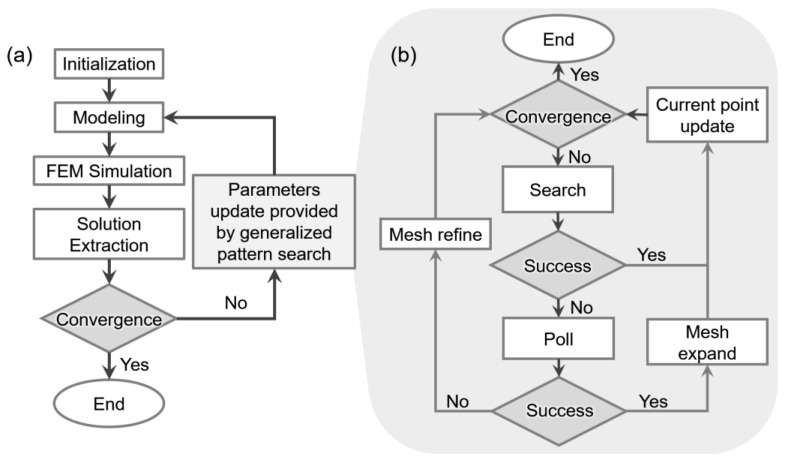
Workflow of the proposed data-driven optimization strategy. (**a**) Overall working mechanism. (**b**) Detailed workflow of generalized pattern search.

The proposed optimization strategy uses FEM simulation as the solver and GPS algorithm as the optimizer, whose workflow is depicted in Figure 2. The initialization step is mainly used for setting parameters in the GPS algorithm, including initial point, the searching and polling method, stopping criteria and other parameters used to accelerate the optimization. Moreover, a new set of geometric parameters generated by GPS is used for FEM modelling, and then, the FEM simulation is performed in order to extract the solutions for feedback to the GPS algorithm. In order to efficiently harvest energy in daily life such as cars, bridges and the human body, the development of PEHs tends to have high output power density and low resonant frequency. Therefore, the optimization objective function here is defined as normalized power density or output power. The definition of normalized power density is shown as Equation (2), which is a function of various geometric parameters and external excitation:

(2) $$P_{n}=\frac{P}{a^{2}\cdot f_{1}\cdot V_{eff}}$$

where $P_{n}$ denotes the normalized power density, P denotes the output power, $f_{1}$ denotes the first-modal resonant frequency, $V_{eff}$ denotes the effective volume of the specific structure, and a represent the excitation acceleration. The maximizing of normalized power density may indicate the trend of maximizing output power and minimizing the first-modal resonant frequency and effective volume. Therefore, the optimization problem is defined as below:

(3) $$\begin{array}{cc} maximize & P_{n}\;or\;P\\ \mathrm{subject}\;\mathrm{to} & 0.5\mathrm{mm}\leq l_{p}\leq 15\;\mathrm{mm}\\ & 0.5\;\mathrm{mm}\leq w\leq 3\;\mathrm{mm}\\ & 0.001\;\mathrm{mm}\leq t_{p}\leq 0.05\;\mathrm{mm}\\ & 0.03\;\mathrm{mm}\leq t_{b}\leq 0.05\;\mathrm{mm} \end{array}$$

## 3. Results and Discussion

**Figure 3:**
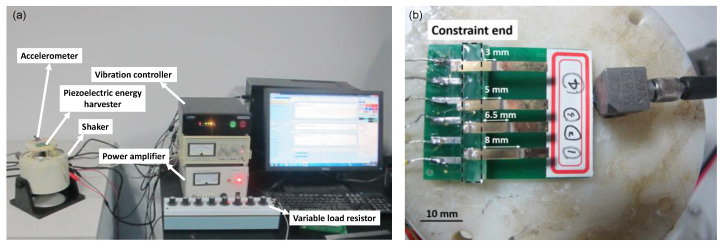
(**a**) Photograph of the experimental setup. (**b**) Photograph of the fabricated harvesters with the varied PZT layer length.

Utilizing the well-described micromachining processes and experimental setup in our previous work [17], we first fabricated and tested four devices with different length PZT layers to validate the effectiveness of the proposed data-driven optimization strategy, and then, individually optimized the PZT length and the proof mass length intending to maximize output power and compared the optimized result with the previous works [18,23]. The experimental setup is shown in Figure 3a. Controlled by the vibration controller through the power amplifier (YE2706A, Sinocera Piezotronics, Inc., Yangzhou, China), the force applied on different PEHs is generated by a shaker (JK-2, Sinocera Piezotronics, Inc.) and is monitored by a force sensor (208C02, PCB Piezotronics, New York, NY, USA).

Firstly, the bulk PZT (300 μm) and beryllium bronze (50 μm) were polished so as to increase the bonding strength. Then, the bulk PZT was bonded on the beryllium bronze using conductive silver epoxy in a vacuum oven at 175 °C for 3.5 h.

**Figure 4:**
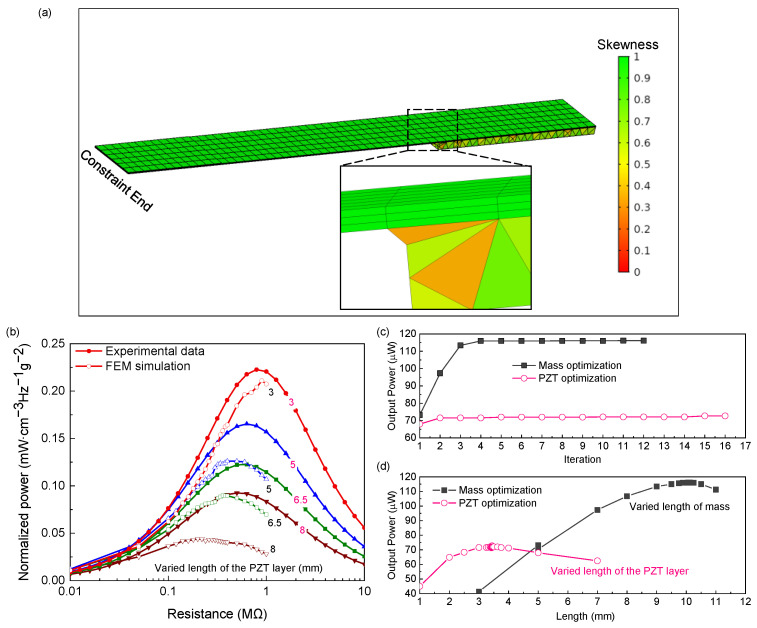
(**a**) The mesh of the PEHs in FEM simulation. Validation of the proposed scheme by (**b**) comparison of normalized output power for the presented PEHs with the varied PZT layer length under different external resistance between FEM simulation and experimental results. (**c**) Convergence curve of output power for each single-variable optimization given by the proposed scheme. (**d**) All results searched by the proposed scheme for each single-variable optimization. The applied acceleration amplitude is 1.0 g.

Next, the bulk PZT was thinned to around 50 μm through chemical mechanical thinning and polishing. After that, 20/200 nm Cr/Au was sputtered on the polished surface of the bulk PZT thick film as an electrode. Finally, the cantilevered PEHs were patterned using the ultraviolet laser method, and the proof mass was assembled at the free end. The fabricated harvesters with different PZT layer length are illustrated in Figure 3b. Under 1.0 g acceleration, the PEHs were connected in serial with the external resistance, which varied from 1 kΩ to 1000 kΩ to determine the optimum load resistance and output power. For performing FEM simulation, a geometrical configuration was considered where the free end was mounted with a proof mass, and the upper surface of the PZT was grounded. In addition, chamfer was added between the proof mass and structural layer to avoid stress singularities at the reentrant corners. Meshes were created according to the shape of the geometry. Then, the detailed meshes were determined by carrying out a mesh convergence study. Figure 4a presents the meshes of the PEHs constructed in COMSOL software. Here, we used skewness, the default quality measure in COMSOL software, to evaluate the mesh quality, which is a suitable measure for most types of meshes. To extract the optimum output power of each PEH, the FEM simulation firstly ran an eigenfrequency study to obtain the first-modal eigenfrequency of the PEHs, and then ran a frequency domain study under the first-modal eigenfrequency with an auxiliary sweep of different external resistance. The excitation was set as body load with an acceleration of 1.0 g (9.8 m/s^2^). Using the proposed scheme, the procedures mentioned above will not stop until the GPS algorithm meets convergence, and the structure of PEHs will keep updated to seek the optimum geometric parameters.

Figure 4b shows a comparison of normalized output power for the presented PEHs with the varied PZT layer length under different external resistance between FEM simulation and experimental results. Compared with the experimental data, the FEM simulation showed agreement in the trend of maximum output power with varied PZT layer length, which proved the validation of the FEM simulation in determining the optimal geometric parameters. Especially when the length of the PZT layer is 3.0 mm, the FEM simulation fits well with the experimental data. The difference in output power corresponding to external resistance between FEM simulation and experimental results may come from manufacturing and testing errors. As the length of the PZT layer increases, the manufacturing errors accumulate. The difference between FEM and the experiment decreases as the length of PZT decreases. Moreover, the mechanical damping (set as 0.015) was assumed to be a constant in the FEM simulation, which may also lead to errors in power prediction for different structure [32]. Then, two single-variable optimizations (PZT length and mass length) for maximum output performance were performed and compared with the previous works [18,23]. For the cantilever harvester with proof mass, Hu et al. [18] and Jia et al. [23] have, respectively, optimized the PZT length and the mass length to maximize output power, from which they have concluded that the optimal ratio of PZT length and total length is approximately 0.2 (3 mm/15 mm), while the optimal mass-beam length ratio is 0.6~0.7.

Figure 4c shows the convergence curve of mass and PZT length optimization. It can be observed that the GPS algorithm can efficiently optimize the proposed optimization problems within two and four iterations, respectively, for PZT length and mass length optimization. Figure 4d presents all results evaluated by the GPS algorithm, which demonstrates the trend of output power versus varied length of the mass and PZT. The optimal PZT length and mass length are 3.437 mm and 10.090 mm corresponding to the ratio of the total length of 0.229 and 0.672, respectively. The agreement of the optimized results and that of the previous works [18,23] proves the efficiency and effectiveness of the proposed data-driven optimization method and the FEM model. Furthermore, unlike the optimization approaches in the previous works [18,23], by which researchers manually varied the geometric parameters at fixed intervals to explore the parameter space for maximum output performance, the proposed method automatically performs the optimization task once set, saving researchers time compared to trial-and-error approaches. In addition, a more precise exploration in the parameter space can be carried out by the proposed scheme compared with the manual trial-and-error approaches. Respectively labeled as 1OPT-1 and 1OPT-2, the geometric parameters and output performance of the above two single-variable optimizations are summarized in Table 2.

After verifying the effectiveness of the proposed scheme, further optimization for the unimorph PEHs was implemented to obtain maximum output power and minimum resonant frequency. The optimization problem is depicted as Equation (3). The geometric parameters to be optimized were set as $l_{p},\;w,\;t_{p}$ and $t_{b}$. As the thickness of the whole beam becomes thinner, the higher the probability of device failure. Based on the experimental data in the previous work [18], the upper and lower bound of $t_{b}$ was set as 30–50 mm from a conservative consideration. The optimal results and the initial point in the optimization, labeled as 4OPT-1 and Ref, are summarized in Table 2.

**Figure 5:**
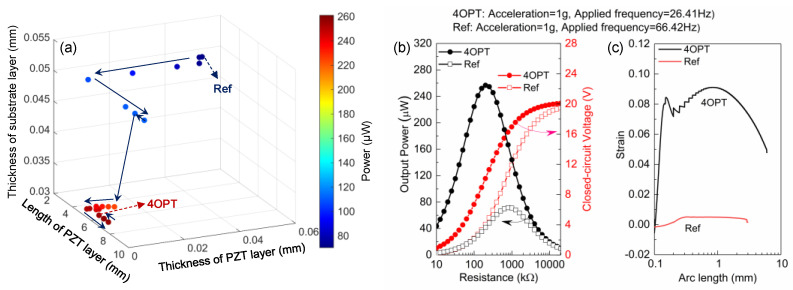
Evaluation of the proposed data-driven optimization strategy from various perspectives. (**a**) Trajectory of the parameters during optimization. (**b**) Output power versus external resistance for previous reported high-output PEH and optimized structure. (**c**) Strain distributions along the arc length of previous reported high-output PEH and optimized structure.

Figure 5a presents the trajectory of the geometric parameters and output power during optimization, which directly shows how the GPS algorithm optimizes the defined optimizable parameters to obtain better values of objectives function. Each point, respectively, represents the best point at every iteration. As shown in Figure 5a and Table 2, the optimal length and thickness of PZT are 6.0 mm and 4.6 μm, respectively. In addition, the reduction in thickness of the structural layer also contributes to the improvement of output power. Figure 5b demonstrates the comparison of the output performance between the reference structure (Ref) and optimized structure (4OPT-1). It can be observed that the resonant frequency of 4OPT-1 is 2.54 times lower than that of Ref, while the output power and normalized power density were, respectively, about 3.71 and 10.10 times larger, showing a substantial improvement in output performance. Numerically, the reduction in resonant frequency greatly contributed to the improvement of normalized power density according to the definition given by Equation (2). By calculating the second derivative of the tip displacement at the first-modal vibration, the strain of the piezoelectric layer along the arc length ($S_{1}\left(x,y,t\right)=-y\frac{\partial^{2}z\left(x,t\right)}{\partial x^{2}}$) is presented in Figure 5c, which demonstrates a higher average strain distribution of 4OPT-1 than that of Ref, leading to an improvement in the output performance of 4OPT-1. However, it is noted that the effects of a reduction in material properties such as piezoelectricity with the decrease in the thickness of PZT are neglected theoretically. Although the output performance of 4OPT-1 may be augmented without the consideration of the effects of material properties, the optimal result (4OPT-1) given by the proposed data-driven optimization strategy provides the trend of improving output performance by tuning geometric parameters.

**Figure 6:**
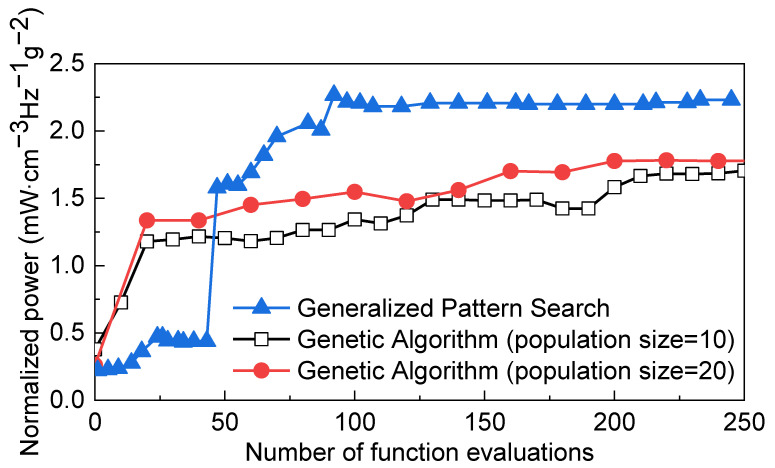
Comparison among the efficiency of generalized pattern search algorithm and genetic algorithm with different population size.

Furthermore, a comparison between the GPS algorithm and GA for the four-variable optimization problem has been implemented and is depicted in Figure 6, which shows the convergence curve versus the number of function evaluations using the GPS algorithm and GA with different population size. The parameters used in GA are the default settings in MATLAB; that is, the mutation method and crossover method are constraint dependent, and the selection method is stochastically uniform. Each configuration was limited to run approximately 250 times for comparison.

To some extent, the number of function evaluations can reflect the running time due to having the same solver (FEM simulation) for each configuration. Although the genetic algorithm can obtain a higher normalized output power at the beginning, the GPS algorithm succeeds in achieving better performance than the GA algorithm with a population size of 10 or 20 after evaluation 250 times. Furthermore, the GPS algorithm requires fewer function evaluation than the GA. The reasons why the pattern search algorithm is more efficient than the GA algorithm with a population size of 10 or 20 are numerous, including the characteristics of the optimization problem, the complex parameter tuning of GA algorithm, and the size of parameter space. Theoretically, the performance of GA may be improved by increasing the population size, whereas the running time may increase for the proposed optimization problem in this work, possibly leading to a low efficiency of optimization.

**Figure 7:**
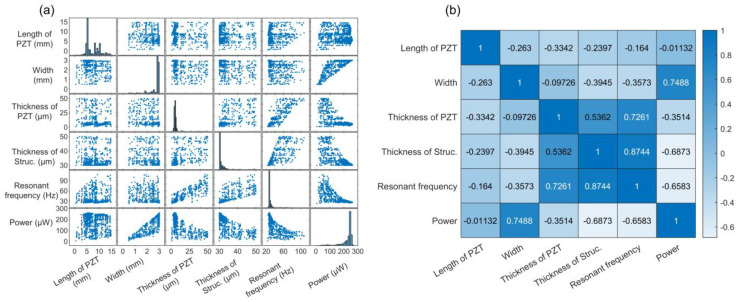
(**a**) Scatterplot and (**b**) Spearman’s rank correlation matrix of the geometric parameters and output performance (resonant frequency and output power).

Figure 7a shows a scatterplot among the geometric parameters $l_{p},\;w,\;t_{p},\;t_{b}$, resonant frequency and output power. It is observed that the data are not completely randomly distributed in the parameter space due to the minimum searching characteristic of the GPS algorithm and GA. The peak in each histogram in the first four rows indicates the preferable geometric parameters given by the GPS algorithm and GA that may generate better output performance. The contour of the scatterplot of the power and thickness of PZT, the power and thickness of the structural layers, and the power and resonant frequency may present non-linearity among the aforementioned variables. Based on the data in the scatterplot, Spearman’s rank correlation coefficient was calculated using Equation (4) to evaluate the strength and direction of the monotonic relationship among the geometric parameters, resonant frequency and output power.

(4) $$r_{s}=1-\frac{6\sum d_{i}^{2}}{n\left(n^{2}-1\right)}$$

where $d_{i}=rg\left(X_{i}\right)-rg\left(Y_{i}\right)$, representing the difference in rank between $X_{i}$ and $Y_{i}$; n is the number of observations; $X_{i}$ and $Y_{i}$ are variables to be evaluated.

The correlation matrix with normalized values is presented in Figure 7b. The greater the absolute value of the correlation coefficient, the stronger the monotonic relationship between the evaluated variables. After filtering the identical data, the amount of data available for calculating the Spearman’s correlation coefficient is 4506. The maximum value in the correlation matrix is 0.8744 and the corresponding *p*-value under t-distribution is 0, suggesting a strong positive correlation between the thickness of the structural layers and resonant frequency, which means that the designers may need to reduce the thickness of the structural layers to lower the resonant frequency. The *p*-value under t-distribution is calculated using Equation (5):

(5) $$t=r_{s}\sqrt{\frac{n-2}{1-r_{s}^{2}}}$$

In addition, the coefficient between power and width is 0.7488, suggesting that the designers may increase the width to improve the output power. However, it is noted that the coefficient of resonant frequency and width is −0.3573, indicating a weak negative correlation between them. Expanding the width may increase the resonant frequency. Designers may need to balance the impact of increase on width. Moreover, although the coefficient of power and length of PZT is close to 0, its *p*-value (0.4475) is larger than the general threshold of 0.05, which indicates that this correlation may fail to reject the null hypothesis. As shown in Figure 7a, although the scatterplot of the power and length of PZT presented no monotonic relationship, the upper contour in the scatterplot showed a non-linearity relationship between them, in which the optimal length of PZT is 6.0 mm when the thickness of PZT is 4.6 μm. In addition, in the scatterplot of the power and the length of PZT, although the length of PZT is set as the optimum (6.0 mm), the output power may encounter poor performance without proper setup of other geometric parameters. Therefore, it is necessary to simultaneously optimize multiple parameters for improving the performance of PEHs using the proposed data-driven optimization strategy.

## 4. Conclusions

In summary, a data-driven optimization strategy based on FEM simulation (solver) and a GPS algorithm (optimizer) was proposed and implemented, which can not only optimize the geometric parameters of PEHs to achieve high performance but also save time for analytical modeling and complex parameter tuning. In addition, the proposed strategy can search the variable space more precisely than manually varied geometric parameters at fixed intervals. The effectiveness and efficiency of the proposed scheme were validated by comparing the optimization results with previous works [18,23] and experimental results. Then, a four-variable ($l_{p},\;w,\;t_{p}$ and $t_{b}$) optimization was further investigated with the aim of maximum output performance. The optimal ratio of the length of PZT and structural layer increases as the thickness of PZT decreases compared with previous works. The optimization results showed that the optimal length ratio is 0.40 (6.0 mm/15.0 mm) when the thickness of PZT is 4.6 µm, while the optimal length ratio is 0.23 (3.4 mm/15.0 mm) when the thickness of PZT is 50 µm. Furthermore, Spearman’s rank correlation coefficient was calculated for evaluating the correlation among geometric parameters and output performance such as resonant frequency and output power, providing a data-based perspective on the design and optimization of piezoelectric energy harvesters.

Our evaluation showed that the GPS algorithm exhibited better performance than GA in terms of efficiency, requiring fewer function evaluations than GA. Solving the multi-parameter coupling problem such as that of PEHs may require expensive computation cost. Therefore, utilizing a GPS algorithm can shorten the total optimization time for complex high-dimensional optimization problems in real life. In addition, owing to the extensive application of the FEM simulation, the proposed strategy is not limited to the optimization of PEHs, but can also be used for the optimization of other structures that are challenging to formulate, facilitating the employment of the proposed strategy in other fields.

## Figures and Tables

**Table 1:** The material properties used in FEM simulation.

Parameters	Young’s Modulus GPa	Density kg/m^3^	Poisson Ratio	Elasticity Matrix GPa	Piezoelectric Coupling Matrix C/m^2^
PZT	-	7500	0.31	{127.205, 80.2122, 127.205, 84.6702, 84.6702, 117.436, 0, 0, 0, 22.9885, 0, 0, 0, 0, 22.9885, 0, 0, 0, 0, 0, 23.4742}	{0, 0, −6.62281, 0, 0, −6.62281, 0, 0, 23.2403, 0, 17.0345, 0, 17.0345, 0, 0, 0, 0, 0}
Beryllium copper	128	8250	0.3	-	-
Tungsten	411	19,350	0.28	-	-

**Table 2:** Geometric parameters and results of our calculations.

Parameters	*l_p_*	*w*	*l_m_*	*t_p_*	*t_b_*	*f*_1_	*P*	*P_D_*	*P_n_*	Runtime
	mm	mm	mm	µm	µm	Hz	µW	mW·cm^−3^	mW·cm^−3^·Hz^−1^·g^−2^	min
Ref [18]	3.000	2.50	5.00	50.00	50.00	66.96	70.28	14.80	0.221	-
1OPT-1	3.437	2.50	5.00	50.00	50.00	70.19	72.70	15.13	0.216	12
1OPT-2	3.000	2.50	10.09	50.00	50.00	81.46	116.17	15.92	0.195	13
4OPT-1	6.001	3.00	5.00	4.625	30.00	26.38	261.02	58.87	2.232	61

*l_b_* = 15.00 mm; *t_m_* = 0.20 mm; Acceleration = 1 g.

## Data Availability

Data are contained within the article.
